# Four-year clinical and echocardiographic outcomes after tricuspid valve replacement using a novel bovine pericardial valve bioprosthesis

**DOI:** 10.1016/j.xjon.2026.101838

**Published:** 2026-05-04

**Authors:** Abdullatif Abo Dan, Mahée Côté, Anya Chabane, Paul Borchiellini, Philippe Demers, Michel Pellerin, Pierre-Emmanuel Noly, Denis Bouchard

**Affiliations:** a Department of Cardiac Surgery, Montreal Heart Institute, Université de Montréal, Montreal, Quebec, Canada; b Faculty of Medicine, Université de Sherbrooke, Sherbrooke, Quebec, Canada

**Keywords:** valvular heart disease, tricuspid valve replacement, bioprosthetic valve, valve durability, structural valve deterioration, surgical outcomes

## Abstract

**Objective:**

To describe our experience and evaluate the clinical outcomes and hemodynamic performance of a new bovine pericardial valve bioprosthesis when used off-label for tricuspid valve replacement (TVR).

**Methods:**

We retrospectively reviewed 82 consecutive patients who underwent 83 TVR procedures (1 patient required early reoperation for paravalvular leak) with the new bioprosthesis between July 2021 and April 2025 at our institution. The mean clinical follow-up was 2.3 ± 1.1 years (94% completed), and the mean echocardiographic follow-up was 2.0 ± 1.1 years (75% completed). The primary end point was bioprosthetic valve dysfunction; secondary end points were survival, perioperative outcomes, major complications, and hemodynamic performance.

**Results:**

Mean age was 66.6 ± 14.0 years; 51.2% (42/82) were female. New York Heart Association class III-IV symptoms were present in 65.2% (45/69). Principal indications were annular dilatation (65.7%, 46/70) and TriClip failure (11.4%, 8/70). Mean Society of Thoracic Surgeons and TRI-SCORE were 6.9 ± 11.4 and 11.8 ± 7.1, respectively. Sternotomy was performed in 41.0% (34/83) and minimally invasive access in 59.0% (49/83). Concomitant procedures included left-sided valvular surgery (47.0%, 39/83) and left atrial appendage closure (26.5%, 22/83). The most common prosthesis size was 33 mm (67.5%, 56/83). As the primary end point, no valve thrombosis was observed and 2.6% (2/77) developed severe central tricuspid regurgitation (TR) requiring percutaneous reintervention. New moderate intraprosthetic TR occurred in 13.3% (6/45) of patients. Thirty-day mortality was 15.9% (13/82). Major complications included new pacemaker implantation (19.3%, 16/83), reoperation for bleeding (7.2%, 6/83), and cerebrovascular events (7.2%, 6/83). Echocardiography showed stable mean gradients (3.2 ± 1.3 mm Hg at discharge; 3.0 ± 1.2 mm Hg at latest follow-up). Otherwise, 1.3% (1/77) underwent redo TVR for early paravalvular leak. Bioprosthetic endocarditis occurred in 5.2% (4/77), all treated conservatively. Actuarial 4-year survival was 63% and freedom from valvular reintervention was 92%.

**Conclusions:**

The new bioprosthesis implanted in the tricuspid position in this high-risk cohort demonstrated stable hemodynamics; however, 2 cases of valvular dysfunction needing reintervention occurred at 2 years, with 6 cases of stable moderate central TR observed at follow-up. These complications highlight the need for longer follow-up to establish durability and guide patient selection and postoperative care.

**Figure:**
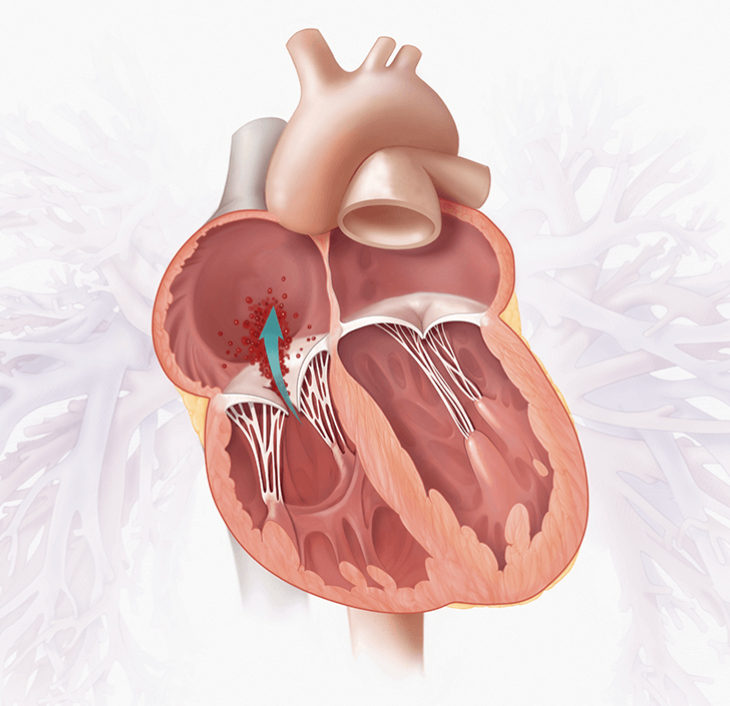
Severe tricuspid regurgitation requiring valve replacement.

**Central Message**

The MITRIS RESILIA mitral prosthesis in the tricuspid position shows persistently low gradients and few early reinterventions.

**Perspective**

In this single-center descriptive retrospective experience, off-label tricuspid replacement with the MITRIS RESILIA mitral bioprosthesis showed favorable early- to midterm hemodynamics and few valve-related reinterventions. Longer follow-up is needed to better define durability.

Tricuspid regurgitation (TR) has emerged as one of the most prevalent yet historically underrecognized valvular disorders, affecting millions of individuals worldwide. Contemporary community-based data demonstrate that moderate-to-severe TR is common in aging populations and is independently associated with progressive right-sided heart failure, recurrent hospitalization, and excess mortality.^1^^,^^2^ As the prevalence of atrial fibrillation (AF), cardiac implantable electronic devices, and left-sided valve interventions increases, the number of patients presenting with advanced TR continues to rise, underscoring the growing public health relevance of this condition.^1^

Current international practice guidelines advocate tricuspid valve repair as the preferred surgical strategy whenever anatomically feasible, particularly during concomitant left-sided valve operations.^3^^,^^4^ Nevertheless, tricuspid valve replacement (TVR) remains unavoidable in substantial subset of patients presenting with severe leaflet tethering, advanced annular dilatation, infective destruction, prosthetic dysfunction, or previous failed repair where durable competence cannot be achieved.^3^, ^4^, ^5^ Despite the increasing recognition of the clinical impact of TR, surgical intervention is frequently performed late in the disease course, often in patients with advanced right-sided heart failure and multiple comorbidities.^5^

Contemporary registry analyses confirm that patients undergoing isolated TVR represent a high-risk cohort with substantial perioperative morbidity and mortality, reflecting both delayed referral and operative complexity.^5^^,^^6^ These realities have intensified interest in strategies that can improve procedural safety while enhancing long-term prosthetic performance in the tricuspid position.

Prosthesis selection in the tricuspid position presents a unique challenge. Although mechanical valves offer theoretical structural durability, lifelong anticoagulation and thrombosis risk in the low-pressure right heart remain significant concerns. Consequently, bioprosthetic valves are frequently favored, although their principal limitation remains structural valve deterioration (SVD).^7^ Advances in tissue-preservation technologies have sought to mitigate calcium-mediated degeneration of bovine pericardium. MITRIS RESILIA tissue technology (Edwards Lifesciences) incorporates a proprietary aldehyde-capping and glycerol-based preservation process designed to reduce calcification and improve durability.^8^ Nevertheless, the implantation of mitral-designed bioprostheses in the tricuspid position has historically been a common surgical practice in the absence of purpose-built tricuspid valves. In this evolving landscape, reporting of real-world surgical TVR outcomes remains essential. We therefore conducted a single-center retrospective evaluation of clinical, echocardiographic, and valve-related outcomes after surgical TVR using the MITRIS RESILIA mitral bioprosthesis in the tricuspid position, with a focus on procedural feasibility, early- to midterm hemodynamic performance, and clinically relevant valve-related events in contemporary practice.

## Methods

### Study Design and Patient Population

We report a single-center retrospective descriptive review of 82 consecutive patients who underwent 83 surgical TVR using the MITRIS RESILIA bovine pericardial mitral bioprosthesis between July 2021 and April 2025 at the Montreal Heart Institute. This bovine pericardial valve is a mitral-labeled prosthesis used off-label in the tricuspid position in this study. The cohort represents an all-comers population reflecting real-world practice, including patients with intermediate-to-high operative risk, previous cardiac surgery, and significant comorbidity burden. Indications for replacement included severe symptomatic TR with unfavorable anatomy for durable repair, including cases in which the treating surgical team judged repair unlikely to provide durable competence on the basis of the overall anatomic and clinical context. In our practice, leaflet tethering greater than 5 mm below the annular plane was considered a concerning feature for repair durability. Accordingly, valve replacement could be selected when this finding was present in the setting of otherwise-unfavorable anatomy. The present study does not provide a formal repair-versus-replacement algorithm. Both first-time surgical patients and redo operations were included, as were patients undergoing concomitant cardiac procedures (mitral valve surgery, aortic valve surgery, coronary artery bypass grafting, or arrhythmia surgery). Shared decision-making with patients was performed in all cases regarding prosthesis type. Given the retrospective observational design, individual patient consent was waived in accordance with institutional guidelines. The study was approved by our local institutional review board (approval #2025-3506) on September 23, 2025.

### Operative Technique

Surgical access was obtained through median sternotomy or minimally invasive video-assisted right minithoracotomy. Standard cardiopulmonary bypass under normothermia was used. Myocardial protection and use of aortic crossclamping were tailored to the operative setting. Aortic crossclamping was not routine for isolated TVR and was primarily used when required by concomitant procedures or specific intraoperative considerations. When cardioplegic arrest was used, myocardial protection was achieved using blood cardioplegia administered antegrade or combined antegrade/retrograde depending on operative context. The novel bovine pericardial mitral prosthesis, packaged dry and not rinsed before implantation, was secured in the tricuspid annulus using interrupted pledgeted everting sutures tied with automated fastening devices. Native leaflet and subvalvular tissue preservation was performed when feasible. In patients undergoing concomitant surgical ablation, the maze procedure was generally performed as a biatrial cryoablation lesion set most closely resembling a Cox-maze IV pattern.

### Postoperative Management

Postoperative antithrombotic strategy was individualized according to patient risk profile and rhythm status. Patients received either vitamin K antagonist therapy or direct oral anticoagulants (DOAC) for 3 months postoperatively in the absence of additional long-term indications, or lifelong anticoagulation when clinically indicated, in accordance with contemporary guideline-based practice and multidisciplinary consensus. Rhythm monitoring and pacemaker implantation were performed as clinically indicated.

### Data Collection and Follow-up

Clinical and echocardiographic data were retrospectively entered into a dedicated institutional database. Follow-up was conducted at specified intervals through outpatient visits and structured telephone interviews for patients unable to attend in person. The most recent available transthoracic echocardiography (TTE) was included for analysis. Clinical and echocardiographic follow-up was considered complete in December 2025. Follow-up duration was calculated and expressed in valve-years.

### Echocardiographic Assessment

Hemodynamic performance was evaluated using serial TTE at discharge and longitudinal follow-up. Measurements included mean and peak transprosthetic tricuspid gradients, tricuspid valvular area, severity of residual or recurrent TR, and presence of prosthetic stenosis, thrombosis, or paravalvular leak (PVL).

### Study End Points

The primary end point was bioprosthetic valve dysfunction (BVD). Secondary end points included overall survival, perioperative outcomes, major complications, and hemodynamic performance. Perioperative outcomes specifically included 30-day mortality, major adverse cardiovascular and cerebrovascular events, and the requirement for permanent pacemaker implantation. Major complications comprised prosthetic valve thrombosis, infective endocarditis, and valve-related reintervention, including surgical reoperation or transcatheter valve-in-valve procedures. Infective endocarditis was included to capture clinically relevant valve-related adverse events during follow-up rather than to test a specific hypothesis regarding differential tissue susceptibility. Hemodynamic performance was assessed by serial TTE, as described previously. BVD and SVD were defined according to Heart Valve Collaboratory and European Association of Percutaneous Cardiovascular Interventions/European Society of Cardiology/European Association for Cardio-Thoracic Surgery consensus definitions, adapted to the tricuspid position.^9^, ^10^, ^11^ Thrombosis detection relied on routine clinical follow-up and TTE, with further investigations performed when clinically indicated. Systematic cardiac computed tomography surveillance for subclinical leaflet thrombosis was not performed.

### Statistical Analysis

Statistical analyses were performed using R, version 4.5.1 (R Foundation for Statistical Computing). Continuous variables are presented as mean ± standard deviation, median, and range, whereas categorical variables are reported as counts and relative frequencies (percentages). Early events (≤30 days postoperatively) are expressed as simple proportions (number of events divided by the total study population). Late events (>30 days) are reported as linearized occurrence rates. Event-free survival was estimated using the Kaplan-Meier method. Follow-up consisted of clinical assessments conducted between July 2025 and December 2025, as well as echocardiographic evaluations performed at hospital discharge (within 30 days after surgery) and during follow-up between July 2024 and December 2025.

## Results

### Baseline Clinical Presentation

A total of 83 TVR procedures were analyzed, with 1 patient undergoing 2 operations in the same week because of a severe paravalvular leak; thus, each surgical procedure was counted as a separate case. The mean age was 66.6 ± 14.0 years, with 31.3% of patients younger than 65 years old. Female sex accounted for 51.8% of the cohort. At baseline, 65.2% of patients (45/69) were in New York Heart Association (NYHA) class III-IV. Available data for etiologies of TR included annular dilatation (65.7%), rheumatic disease (2.9%), endocarditis (8.6%), subvalvular apparatus rupture (1.4%), congenital disease (5.7%), TriClip failure (11.4%), TR secondary to pacemaker lead (1.4%), and prosthetic valve dysfunction (1.4%). No cases of ischemic TR were reported. Subvalvular apparatus rupture included papillary rupture (n = 1) and flail (n = 1). TR severity at baseline was predominantly severe (97.5%), with mild in 2.5% of cases. TR grade alone was not the indication for replacement. The surgical indications for mild TR included mechanical prosthetic thrombosis and vegetations on the tricuspid annulus in the context of mitral-tricuspid infective endocarditis. Comorbidities were frequent: hypertension (84.3%), dyslipidemia (74.4%), coronary artery disease (22.9%), diabetes (27.7%), and end-stage renal disease (3.6%). AF was present in 79.5% of patients. Among patients with AF, 42.4% had paroxysmal AF, 33.3% had persistent AF, and 24.2% had long-standing persistent AF. This classification was used descriptively within this retrospective review and was not intended to imply a specific heart-team strategy regarding rate control, rhythm control, or anticoagulation. The mean Society of Thoracic Surgeons (STS) predicted mortality score was 6.9% ± 11.4%, with a mean TRI-SCORE predicted mortality of 11.8% ± 7.1%. Baseline characteristics of the population are summarized in Table 1.

**Table 1:** Baseline clinical and demographic characteristics

Variable	% (n) or mean 土 SD
Age	
Age, y	66.6 ± 14.0
Patients <65 y	30.1% (25/83)
Female sex	50.6% (42/83)
NYHA classification III-IV	65.2% (45/69)
TRI-SCORE predicted mortality	11.8% ± 7.1%
Etiology	
Annular dilatation	65.7% (46/70)
Rheumatic disease	2.9% (2/70)
Infective endocarditis	8.6% (6/70)
Subvalvular apparatus rupture	1.4% (2/70)
Congenital disease	5.7% (4/70)
TriClip failure	11.4% (8/70)
Pacemaker lead extraction	1.4% (1/70)
Prosthetic valve dysfunction	1.4% (1/70)
Tricuspid regurgitation	
0 (none)	0.0% (0/81)
1 (mild)	2.5% (2/81)
2 (moderate)	0.0% (0/81)
3 (severe)	97.5% (79/81)
Comorbidities	
Diabetes	27.7% (23/83)
Hypertension	84.3% (70/83)
Dyslipidemia	73.5% (61/83)
Coronary artery disease	22.9% (19/83)
End-stage renal disease	3.6% (3/83)
STS score	6.9% ± 11.4%
AF (total)	79.5% (66/83)
Paroxysmal	42.4% (28/66)
Persistent	33.4% (22/66)
Long-standing persistent	24.2% (16/66)

Values are presented as mean ± SD or percentage. One patient underwent 2 operations in the same year; each surgical procedure (including the patient's clinical status at that time) was counted as separate cases. Denominators vary by variable because of missing data. *SD*, Standard deviation; *NYHA*, New York Heart Association; *STS*, Society of Thoracic Surgeons; *AF*, atrial fibrillation.

### Surgical Intervention

TVR was performed via minimally invasive access in 59.0% of cases and through median sternotomy in 41.0%. Most procedures were elective (84.3%), with urgent (13.3%) and emergent (2.4%) operations performed less frequently. Cardiopulmonary bypass time averaged 115.2 ± 52.7 minutes, with an aortic crossclamp mean time of 73.1 ± 40.6 minutes. The reported mean aortic crossclamp time should not be interpreted as representative of isolated TVR alone. In our practice, aortic crossclamping was not routine for isolated TVR, as 12 isolated cases were performed without crossclamping. Therefore, the observed mean crossclamp time largely reflects procedures involving concomitant cardiac surgery or other intraoperative indications for cardioplegic arrest. Isolated TVR was performed in 48.2% of patients. Concomitant procedures were undertaken in 51.8% of cases and included left-sided valvular surgery (47.0%), aortic valve replacement (13.3%), coronary artery bypass grafting (10.8%), maze cryoablation (22.9%), left atrial appendage exclusion (26.5%), and other cardiac procedures (13.3%). The most frequently implanted prosthesis size was 33 mm (67.5%), followed by 31 mm (24.1%), 29 mm (7.2%), and 27 mm (1.2%). Operative characteristics are seen in Table 2.

**Table 2:** Operative characteristics

Variable	% (n) and mean 土 SD
Surgical technique	
Open	41.0% (34/83)
Minimally invasive	59.0% (49/83)
Surgical timing	
Elective	84.3% (70/83)
Urgent	13.3% (11/83)
Emergent	2.4% (2/83)
Cardiopulmonary bypass time, min	115.2 ± 52.7
Crossclamp time, min	73.1 ± 40.6
Isolated TVR	48.2% (40/83)
Concomitant procedures	51.8% (43/83)
Left-sided valvular surgery	47.0% (39/83)
Aortic valve replacement	13.3% (11/83)
Coronary artery bypass grafting	10.8% (9/83)
Cryoablation lesions	22.9% (19/83)
Left atrial appendage closure	26.5% (22/83)
Other cardiac procedures	13.3% (11/83)
Tricuspid valve prosthesis size	
25 mm	0.0% (0/83)
27 mm	1.2% (1/83)
29 mm	7.2% (6/83)
31 mm	24.1% (20/83)
33 mm	67.5% (56/83)

Values are presented as mean ± SD for continuous variables and as percentage (n/N) for categorical variables. Percentages (n/N) represent the proportion of each variable relative to the number of total procedures. Aortic crossclamping was not routine for isolated TVR and primarily reflected cases requiring concomitant procedures or specific intraoperative indications. *SD*, Standard deviation; *TVR*, tricuspid valve replacement.

### In-Hospital Outcomes

Thirty-day mortality occurred in 15.9% (13/82) of patients. Major postoperative events included reoperation for bleeding in 7.2% (6/83) and stroke in 7.2% (6/83). No cases of prosthetic valve thrombosis were observed. Otherwise, 1.3% (1/77) underwent redo TVR for early paravalvular leak. Respiratory complications included pneumonia in 10.8% (9/83), tracheostomy in 3.6% (3/83), and reintubation in 3.6% (3/83). Wound infection occurred in 2.4% (2/83). Electrophysiologic events were frequent, with postoperative AF in 28.9% (24/83) and new permanent pacemaker implantation (epicardial or leadless) in 19.3% (16/83). Additional complications included dialysis in 14.5% (12/83) and phrenic nerve injury in 3.6% (3/83). A summary of in-hospital outcomes is presented in Table 3.

**Table 3:** Postoperative events

Variable	Postoperative events, %
Major events	
30-d mortality	15.9% (13/82)
Reoperation for bleeding	7.2% (6/83)
Stroke	7.2% (6/83)
Thrombosis	0.0% (0/83)
Early complications	
Tracheostomy	3.6% (3/83)
Reintubation	3.6% (3/83)
Pneumonia	10.8% (9/83)
Wound infection	2.4% (2/83)
Electrophysiologic disorders	
Permanent pacemaker implantation	19.3% (16/83)
POAF	28.9% (24/83)
Nerve injuries and renal complications	
Phrenic nerve injury diagnosis	3.6% (3/83)
Dialysis	14.5% (12/83)

Percentages (n/N) represent the proportion of each event relative to the number of total procedures, except for 30-day mortality, as the patient who underwent two procedures had them both within 30 days. Reoperation for bleeding included interventions for active hemorrhage or for the treatment/prevention of cardiac tamponade. *POAF*, Postoperative atrial fibrillation.

### Follow-up Clinical Outcomes

Clinical follow-up was obtained in 77 of 82 patients (93.9%) between July and December 2025, with a mean time between surgery and follow-up of 2.3 ± 1.1 years. All-cause mortality at follow-up was 26.8% (22/82). Among patients with available follow-up, readmission for any cause occurred in 28.6% (22/77). Among the 77 patients with a complete follow-up, 2 (2.6%) developed a new severe TR requiring percutaneous reintervention 2 years after the surgery. Those 2 patients received oral anticoagulation postoperatively. Bioprosthetic endocarditis occurred in 5.2% (4/77), and all cases were treated with intravenous antibiotic therapy alone without surgical or transcatheter reintervention. Major bleeding and stroke were reported in 5.2% (4/77) and 1.3% (1/77), respectively. At last follow-up, 11.7% (9/77) of patients were in NYHA class III-IV. Antithrombotic therapy consisted of oral anticoagulation in 63.6% (49/77) and antiplatelet therapy with aspirin in 6.5% (5/77). Among anticoagulated patients, 40.8% (20/49) received warfarin and 59.2% (29/49) received DOAC. A summary of follow-up clinical outcomes is presented in Table 4.

**Table 4:** Follow-up clinical characteristics

Variable	Follow-up, %
Mortality	
Overall mortality	26.8% (22/82)
Clinical and valvular outcomes	
Readmission	28.6% (22/77)
Valve-related reintervention	2.6% (2/77)
Prosthetic infective endocarditis	5.2% (4/77)
Bleeding	
Major bleeding	5.2% (4/77)
Stroke	1.3% (1/77)
Anticoagulation	63.6% (49/77)
Coumadin	40.8% (20/49)
DOAC	59.2% (29/49)
Antiplatelet	
Aspirin	6.5% (5/77)
Symptomatology	
NYHA III-IV	11.7% (9/77)

One patient underwent 2 operations in the same year; all relevant follow-up information for this patient was collected once after the second surgery. Follow-up was conducted between July and November 2025. Mortality is reported as percentage and fraction among patients with available information (n = 77). *DOAC*, Direct oral anticoagulant; *NYHA*, New York Heart Association.

### Survival and Freedom From Reintervention

**Figure 1:**
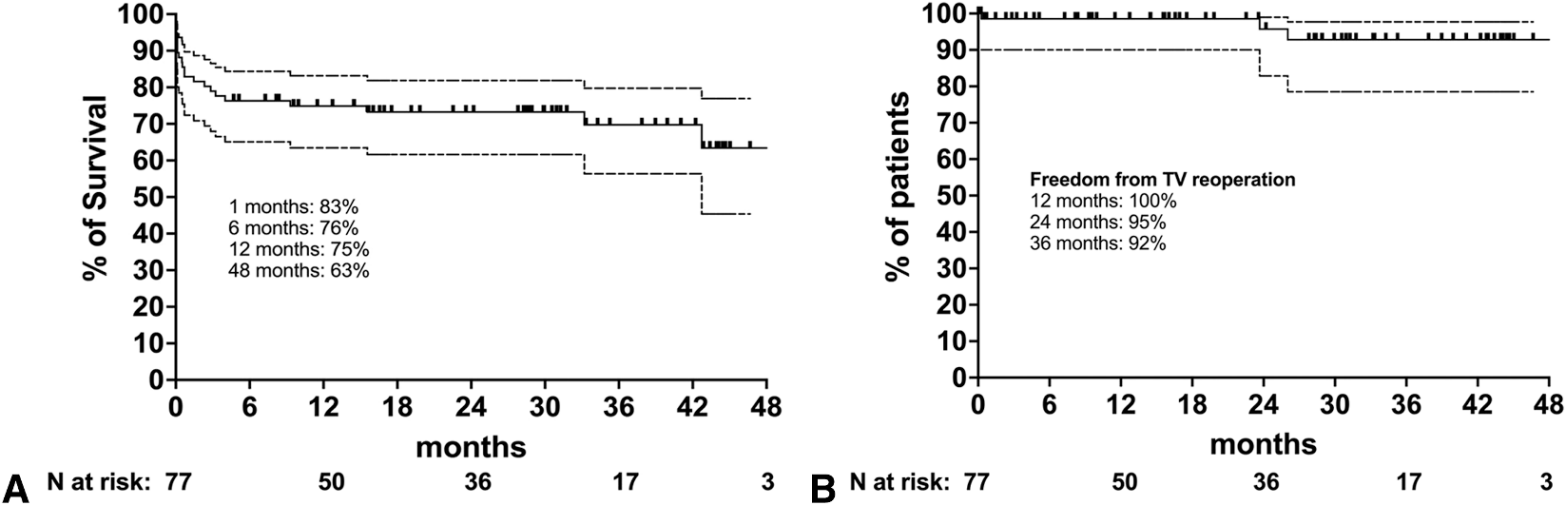
Kaplan-Meier estimates of overall survival and freedom from tricuspid valve (*TV*)-related reintervention after TV replacement with the MITRIS RESILIA bioprosthesis. A, Overall survival: 1 month (95% CI, 72.7%-89.8%), 6 months (95% CI, 65.5%-84.6%), 12 months (95% CI, 63.9%-83.4%), and 48 months (95% CI, 45.6%-77.2%). B, Freedom from tricuspid valve reintervention: 12 months (95% CI, 90.4%-100%), 24 months (95% CI, 83.0%-99.0%), 36 months (95% CI, 78.6%-97.8%), and 48 months (95% CI, 78.6%-97.8%).

Kaplan-Meier estimated overall survival after TVR was 83% at 1 month, 76% at 6 months, 75% at 12 months, and 63% at 48 months (Figure 1). After 1 early reoperation due to PVL, freedom from tricuspid valve–related reintervention was 100% at 12 months, 95% at 24 months, and 92% at 36 and 48 months (Figure 1). Numbers at risk were 77 at baseline, 50 at 12 months, 36 at 24 months, 17 at 36 months, and 3 at 48 months.

### Postoperative Echocardiographic Assessment

Postoperative TTE performed within 30 days of surgery demonstrated a mean left ventricular ejection fraction of 53.2 ± 11.0%, with a left ventricular end-diastolic diameter of 48.5 ± 9.3 mm and end-systolic diameter of 33.9 ± 11.1 mm. Hemodynamic assessment of the tricuspid prosthesis showed a mean transprosthetic gradient of 3.2 ± 1.3 mm Hg and a maximum or peak velocity (Vmax) of 1.5 ± 0.3 m/s. TR was absent in 78.7% (59/75), mild in 18.7% (14/75), moderate in 1.3% (1/75), and severe in 1.3% (1/75) patients. The single early severe regurgitation case was attributable to a PVL, and the patient underwent surgical reintervention 1 week after the index procedure.

### Longitudinal Echocardiographic Follow-up

Follow-up TTE was performed between July 2024 and December 2025. Of the 60 patients alive at follow-up, 45 (75.0%) had an available echocardiographic study. At the latest assessment, left ventricular ejection fraction was 55.0 ± 10.0%, with a left ventricular end-diastolic diameter of 47.0 ± 8.7 mm and left ventricular end-systolic diameter of 31.4 ± 12.4 mm. The mean transprosthetic tricuspid gradient was 3.0 ± 1.2 mm Hg, and Vmax was 1.4 ± 0.5 m/s. Residual TR was absent in 26.7% (12/45), mild in 55.6% (25/45), moderate in 13.3% (6/45), and severe in 4.4% (2/45). Aside from the PVL described postoperatively, the remaining regurgitation cases were intraprosthetic in origin. The 2 severe TR cases observed at follow-up were identified in early 2025, occurring at a mean interval of approximately 2 years after surgery, and were treated with transcatheter tricuspid valve-in-valve replacement. These 2 cases were attributable to intrinsic leaflet degeneration and met criteria for SVD. The 6 moderate central regurgitation cases remain stable under medical management with serial echocardiographic surveillance and no structural leaflet degeneration. All 8 patients received postoperative anticoagulation. Preoperative anticoagulation status was unavailable. None had significant baseline or postoperative comorbidities, such as dialysis-dependent renal disease. The mean interval between surgery and follow-up echocardiography was 2.0 ± 1.1 years. A detailed summary of echocardiographic parameters is provided in Table 5.

**Table 5:** Postoperative and longitudinal changes in transthoracic echocardiographic parameters

Parameter	Postoperative	Follow-up
EF, %	53.2 ± 11.0	55.0 ± 10.0
DD, mm	48.5 ± 9.3	47.0 ± 8.7
SD, mm	33.9 ± 11.1	31.4 ± 12.4
Tricuspid valve		
Mean gradient, mm Hg	3.2 ± 1.3	3.0 ± 1.2
Vmax, m/s	1.5 ± 0.3	1.4 ± 0.5
Tricuspid regurgitation		
0 (None)	78.7% (59/75)	26.7% (12/45)
1 (Mild)	18.7% (14/75)	55.6% (25/45)
2 (Moderate)	1.3% (1/75)	13.3% (6/45)
3 (Moderate-to-severe)	0.0% (0/75)	0.0% (0/45)
4 (Severe)	1.3% (1/75)	4.4% (2/45)

Values are presented as mean ± standard deviation or as percentages (n/N). Postoperative values reflect assessments within 30 days after surgery; follow-up values correspond to TTE done between July 2024 and December 2025. Cohort size varied by parameter due to mortality and data availability. *EF*, Ejection fraction; *DD*, left ventricular end-diastolic diameter; *SD*, left ventricular end-systolic diameter; *TTE*, transthoracic echocardiography.

## Discussion

This contemporary single-center experience evaluating surgical TVR with the MITRIS RESILIA mitral bioprosthesis implanted in the tricuspid position is descriptive in nature but provides several clinically relevant observations. First, survival outcomes in this cohort appear to have been influenced largely by baseline patient risk and operative complexity rather than early prosthetic dysfunction. Second, early- to midterm hemodynamic performance was consistently favorable across valve sizes, with persistently low gradients and preserved forward flow. Third, valve-related adverse events, including thrombosis, major bleeding, and reintervention, were infrequent within the available follow-up. Fourth, although SVD was uncommon, the occurrence of 2 clinically significant intraprosthetic failures requiring transcatheter valve-in-valve therapy highlights important mechanistic and patient-selection considerations in the tricuspid position. Finally, because follow-up duration and imaging completeness were limited, conclusions regarding durability should remain cautious.

### Survival Considerations and Timing of Intervention

The present cohort reflects the well-recognized high-risk phenotype of patients referred for surgical TVR. Thirty-day mortality of 15.9% (13/82) and overall mortality of 26.8% (22/82) at a mean follow-up of 2.3 ± 1.1 years occurred in a population characterized by advanced symptom burden (65.2% NYHA III-IV), high prevalence of AF (79.5%), and intermediate-to-high operative risk (mean STS 6.9% ± 11.4%; TRI-SCORE predicted mortality 11.8% ± 7.1%). Kaplan-Meier survival estimates of 83% at 1 month, 75% at 12 months, and 63% at 48 months demonstrate an early mortality phase followed by relative stabilization, a pattern consistent with contemporary surgical TVR series and reflective of perioperative vulnerability rather than prosthesis-related failure.^3^^,^^12^, ^13^, ^14^ Importantly, early mortality in this cohort occurred in the absence of prosthetic thrombosis and with stable early hemodynamics, underscoring that survival after TVR is primarily determined by the severity of right-sided heart failure, comorbidity burden, and operative complexity rather than intrinsic BVD. On descriptive review, 10 of 13 early deaths occurred in redo operations, 11 of 13 involved at least a concomitant mitral valve replacement, and 11 of 13 occurred in patients with a TRI-SCORE predicted mortality of 14% or greater. These observations help contextualize the observed mortality but should be interpreted cautiously, as no risk-adjusted analysis was performed and independent predictors cannot be established from this dataset. These findings reinforce the growing consensus that earlier referral and less complex operations may yield greater survival benefit than prosthetic design improvements alone.^3^^,^^12^ The cohort's high mean TRI-SCORE (11.8% ± 7.1%) reveals a highly comorbid population and explains the high mortality rate, aligning with contemporary use of dedicated tricuspid surgical risk models. This score has demonstrated predictive validity for in-hospital mortality and is increasingly incorporated into heart-team deliberations and shared decision-making.^15^^,^^16^

### Thromboembolic and Bleeding Risk

No cases of prosthetic valve thrombosis were observed, despite a cohort enriched for AF. However, anticoagulation exposure remained substantial (63.6% on oral anticoagulation at follow-up: 59.2% DOAC, 40.8% warfarin), largely driven by the high prevalence of AF. In-hospital stroke occurred in 7.2%, with 1.3% at follow-up, whereas major bleeding was reported in 5.2%. These event rates fall within the lower range of contemporary surgical TVR reports and support the practical advantage of tissue prostheses in the tricuspid position when balanced against the risks of thrombosis and hemorrhage.^3^^,^^13^^,^^17^

Although the 2 patients who ultimately required reintervention were anticoagulated postoperatively, adherence and international normalized ratio data were unavailable, preventing definitive conclusions regarding subtherapeutic exposure. Nevertheless, the absence of known thrombotic events is concordant with evolving practice patterns, suggesting that anticoagulation may provide additional protective benefit in patients with tissue prostheses, especially when AF is present.^3^^,^^17^^,^^18^

### Hemodynamic Performance and Valve Size Considerations

Early and longitudinal echocardiographic assessment demonstrated consistently low mean transprosthetic gradients and stable forward flow. Within 30 days postoperatively, the mean gradient was 3.2 ± 1.3 mm Hg with Vmax 1.5 ± 0.3 m/s, remaining stable at follow-up (3.0 ± 1.2 mm Hg, Vmax 1.4 ± 0.5 m/s). These values are physiologically reassuring for the tricuspid position and compare favorably with previous surgical TVR reports.^13^^,^^14^

Valve sizing likely contributed to these favorable hemodynamics. The predominance of 31-mm and 33-mm prostheses (91.6% combined) reflects deliberate oversizing relative to annular dilatation and may have minimized patient-prosthesis mismatch in a circulation characterized by high venous return and low transvalvular pressure gradients. The absence of progressive gradient elevation over time further supports early structural stability of leaflet motion and annular conformity. In contrast to left-sided bioprostheses, in which calcific stenosis is a common failure mode, right-sided prosthetic degeneration more frequently manifests through regurgitant mechanisms, a pattern observed in our cohort.^13^

At longitudinal follow-up, most residual TR was mild and hemodynamically stable and insignificant. The moderate central regurgitation cases remained clinically stable under medical management and serial TTE surveillance, without associated progressive gradient elevation during the available follow-up. These findings suggest that gradients alone may not fully capture prosthetic tricuspid valve dysfunction and that longitudinal imaging should also carefully assess regurgitation mechanism and severity.

### Structural Valve Durability and Reoperation

SVD was uncommon but clinically meaningful. Two patients (2.6% of those with follow-up) developed severe intraprosthetic TR at a mean interval of approximately 2 years after index surgery, meeting consensus criteria for SVD and requiring transcatheter tricuspid valve-in-valve replacement.^9^, ^10^, ^11^ Both patients were receiving postoperative anticoagulation, and neither had pacemaker leads, dialysis-dependent renal disease, or other major comorbidities typically associated with accelerated bioprosthetic degeneration. The remaining cases of moderate intraprosthetic regurgitation (6/45 echocardiographic follow-ups, 13.3%) remained stable under surveillance without evidence of leaflet structural disruption. Moderate central regurgitation cases remained clinically stable under surveillance, suggesting possible geometric or functional mechanisms related to the complex right-sided environment rather than definite structural leaflet failure. Freedom from valve-related reintervention remained high (100% at 12 months, 95% at 24 months, 92% at 36 months) after the initial case of early PVL and subsequent novel bovine pericardial mitral bioprosthesis TVR redo. The second implanted valve showed great clinical and hemodynamic outcomes.

The reasons for early intraprosthetic failure in the tricuspid position are likely multifactorial. Although thrombosis was not documented, subclinical leaflet thrombosis or episodic subtherapeutic anticoagulation cannot be excluded in the absence of adherence and INR surveillance.^19^, ^20^, ^21^ In addition, hemodynamic factors unique to the right heart, including chronic volume overload, annular dilatation, and fluctuating venous pressures, may predispose to leaflet stress fatigue and progressive coaptation loss rather than calcific stenosis. Patient-prosthesis geometric interaction may also contribute, with continued annular remodeling or asymmetric dilatation altering leaflet kinematics over time.^3^^,^^22^ Finally, biologic variability in host tissue response, low-grade inflammatory states, or occult infection, despite the absence of overt endocarditis in these 2 cases, remain plausible contributors. The relatively short time to failure argues against classic calcium-mediated degeneration as the primary mechanism and instead supports leaflet loss of integrity as a cause of early SVD.^9^^,^^23^ Given the limited number of events and the descriptive nature of this study, the mechanisms underlying recurrent intraprosthetic regurgitation can only be considered hypotheses rather than definitive conclusions. Although the present dataset does not support strong prescriptive recommendations, our experience suggests that careful attention to patient-specific right-sided anatomy, annular tissue quality, prosthesis sizing, valve seating, and structured follow-up may be particularly important in patients at risk of recurrent dysfunction. Nonetheless, the successful management of both cases with transcatheter valve-in-valve therapy highlights the availability of less invasive reintervention strategies.^24^, ^25^, ^26^

### Patient-Centered Valve Selection

The present findings emphasize that prosthesis choice in the tricuspid position should remain patient-centered and context-dependent. The choice between tricuspid repair and replacement in this cohort was individualized and determined on the basis of the overall anatomic and clinical context rather than annular dilatation alone. In our practice, replacement was considered when repair was judged unlikely to provide durable competence, particularly in the setting of unfavorable anatomy such as marked leaflet tethering. Accordingly, the present study should not be interpreted as proposing a formal repair-versus-replacement algorithm.

Although mechanical valves offer theoretical structural longevity, the cumulative burden of lifelong anticoagulation and heightened thrombosis risk in the low-pressure right heart frequently outweigh durability advantages. Conversely, advanced tissue bioprostheses provide favorable early hemodynamics, lower thrombogenicity, and compatibility with transcatheter valve-in-valve rescue. These attributes are particularly relevant in older and comorbid populations such as the present cohort.^3^^,^^12^^,^^27^ It is also important to acknowledge the temporal evolution of our institutional treatment pathways. During the early study period, percutaneous tricuspid repair options were limited and less frequently used, resulting in a greater proportion of high-risk patients being directed toward surgical intervention. As our transcatheter tricuspid program expanded over time, patients with elevated surgical risk profiles and greater TRI-SCORES were increasingly referred to the percutaneous route, reflecting a progressive shift in case selection.

Within this broader clinical and procedural context, additional considerations emerge when evaluating long-term patient outcomes. In this study, pacemaker implantation and phrenic nerve injury were analyzed descriptively only. Permanent pacemaker new implantations occurred in 19.3% (16/83), reflecting the intrinsic conduction vulnerability of tricuspid surgery and representing an important determinant of postoperative quality of life. Notably, one half of these cases occurred in the setting of redo surgery (8/16), and a similar proportion (9/16) were associated with concomitant cardiac procedures, with only 2 first-time surgery patients (2/16) undergoing an isolated TVR, underscoring the contribution of surgical complexity and cumulative septal manipulation rather than prosthesis-related factors alone. Most cases received a 33-mm prosthesis, but this valve size was also the most commonly implanted overall. Because of the descriptive design, small number of events, and absence of adjusted analysis, these findings should not be interpreted as evidence of independent associations. Phrenic nerve injury was rare (3 cases), and no reliable conclusion can be drawn regarding its relationship to surgical approach or procedural complexity. Nonetheless, contemporary datasets consistently report elevated pacemaker rates after TVR relative to repair, reinforcing the importance of incorporating conduction risk into preoperative counseling and shared decision-making.^28^^,^^29^

### Limitations

This study has several limitations inherent to its design. First, it represents a single-center retrospective observational experience, which limits generalizability and prevents direct comparison with other prosthesis types. Second, there was no comparator group, preventing assessment of relative performance versus other prostheses or surgical strategies. Third, no risk-adjusted analysis was performed despite the reporting of STS score and TRI-SCORE. Therefore, independent determinants of mortality and valve-related events cannot be established. Fourth, although the cohort reflects real-world all-comers practice, the sample size remains modest and the number of valve-related adverse events was low, restricting statistical power for subgroup analyses and multivariable adjustment. Fifth, follow-up duration, while extending to 4 years in a minority of patients, remains predominantly early term to midterm and therefore insufficient to fully characterize long-term structural valve durability in the tricuspid position. Sixth, longitudinal echocardiographic follow-up was available in 45 of 60 surviving patients (75.0%), which may still have led to underestimation of prosthetic valve dysfunction. Seventh, anticoagulation adherence and international normalized ratio variability data were unavailable, preventing definitive assessment of the relationship between anticoagulation exposure and valve-related events. Eighth, thrombosis detection relied on routine clinical follow-up and TTE, and systematic cardiac computed tomography was not performed; accordingly, subclinical leaflet thrombosis may have been underdetected. Finally, the use of an off-label mitral-labeled bioprosthesis in the tricuspid position introduces additional heterogeneity in patient selection and procedural decision-making that may not be reproducible across institutions. Despite these limitations, the study provides important contemporary clinical and hemodynamic data in a population that remains underrepresented in prospective trials. Future multicenter studies with more patients, longer follow-up, complete echocardiographic surveillance, and comparative analyses are needed to definitively establish the role of MITRIS RESILIA mitral valve in contemporary TVR practice.

## Conclusions

In this contemporary single-center retrospective experience, surgical TVR using the MITRIS RESILIA mitral bioprosthesis in the tricuspid position was feasible and associated with favorable early- to midterm hemodynamic performance and a low rate of valve-related reintervention in a high-risk population. However, recurrent intraprosthetic regurgitation occurred in a small number of patients, and conclusions regarding durability remain limited by follow-up duration and imaging completeness. Longer-term multicenter data are needed to better define durability, patient selection, and postoperative surveillance strategies.

### Webcast

You can watch a Webcast of this AATS meeting presentation by going to: https://www.aats.org/resources/four-year-clinical-and-echocar-12239.

**Figure:**
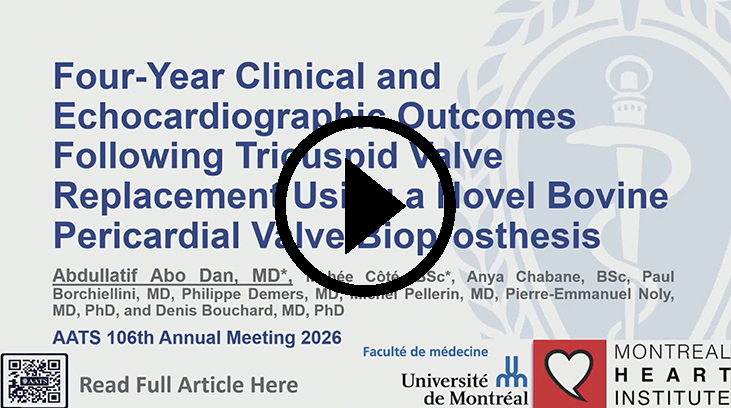

## Conflict of Interest Statement

D.B. received consulting or proctoring fees from Edward Lifesciences and AtriCure. All other authors reported no conflicts of interest.

The *Journal* policy requires editors and reviewers to disclose conflicts of interest and to decline handling or reviewing manuscripts for which they may have a conflict of interest. The editors and reviewers of this article have no conflicts of interest.
